# Comparative genomics approaches to identify genomic regions associated with the antimicrobial activity of Pseudomonas protegens PBL3

**DOI:** 10.1099/mgen.0.001827

**Published:** 2026-09-03

**Authors:** Shilu Dahal, Chia Sin Liew, Jean-Jack Riethoven, Clemencia M. Rojas

**Affiliations:** 1 Department of Plant Pathology, University of Nebraska-Lincoln, Lincoln, NE 68583, USA; 2 Center for Plant Science Innovation, University of Nebraska-Lincoln, Lincoln, NE 68588, USA; 3 Nebraska Center for Biotechnology, University of Nebraska-Lincoln, Lincoln, NE 68588, USA

**Keywords:** antimicrobial gene clusters, *Burkholderia glumae* control, comparative genomics, genome mining, *Pseudomonas protegens *PBL3, secondary metabolites

## Abstract

The environmental bacterium *Pseudomonas protegens* PBL3 has antagonistic activity against the plant pathogenic bacterium *Burkholderia glumae*, an important pathogen in rice. The antimicrobial activity of *P. protegens* PBL3 was found in the bacteria-free secreted fraction (secretome), but the specific molecules, as well as the genetic basis of that activity, have not been identified. In this study, we integrated genomic information with antimicrobial assays on *P. protegens* PBL3 and additional six *Pseudomonas* spp. strains, to identify putative genomic regions in *P. protegens* PBL3 associated with antimicrobial activity. We hypothesized that *Pseudomonas* spp. strains with antimicrobial activity against *B. glumae* have conserved genes with *P. protegens* PBL3 that are absent in strains lacking activity. Comparative genomics analyses with anvi’o and progressiveMauve, and using *P. protegens* PBL3 as the reference genome, revealed 188 genes uniquely present in antimicrobial-producing strains. Seven of those genes were annotated as biosynthetic gene clusters predicted to encode secondary metabolites; additional genes were grouped into 25 contiguous clusters with functions annotated as secretion, signal transduction, regulation, transport/efflux, carbohydrate metabolism and one with an additional uncharacterized function. Altogether, this study uncovered a complex and multi-functional network of candidate genes, suggesting that the antimicrobial activity in *P. protegens* PBL3 is not limited to biosynthetic pathways but also involves additional regulatory, metabolic and export modules to synthesize and deploy antimicrobials.

**Impact Statement**

Bacterial plant diseases can be controlled through biological control, a strategy wherein beneficial micro-organisms known as biological control agents (BCAs) interfere with the biological activities of pathogens through several mechanisms. One of these mechanisms is antibiosis, by which antimicrobial molecules produced by the BCA prevent pathogen multiplication or actively kill the pathogen. While this mechanism of antibiosis has been widely recognized, the specific molecules responsible for the antimicrobial activity are not always identified, given their diverse and complex chemical structures, as well as the unique and intricate biosynthetic pathways. This study unravelled candidate genes and genetic clusters associated with the antimicrobial activity in the environmental bacterium *Pseudomonas protegens* PBL3 to help advance the discovery of effective antimicrobials to control bacterial plant diseases.

## Data Summary

Genome IDs of all eight *Pseudomonas* spp. analysed in this study were obtained from NCBI and JGI Integrated Microbial Genomes and Microbiomes and are listed in Table 1. All supporting data, software versions and protocols have been provided in the methods section or in supplementary data files. Custom analysis code for post-processing anvi'o pangenomics and progressiveMauve whole-genome alignment outputs, along with anonymized example data for testing, is publicly available on GitHub (https://github.com/chiasinL/pseudomonas-project-mgen) and archived on Zenodo (https://doi.org/10.5281/zenodo.19617017).

## Introduction

Plant diseases can be effectively controlled by the biological activities of beneficial micro-organisms in the ecosystem, an approach called biological control [1]. Biological control agents (BCAs) interfere with the activities of plant pathogens either indirectly, by inducing plant resistance or by competing for nutrients or space with pathogens, or directly, by antagonism through the production of antimicrobial molecules that kill or reduce the growth of pathogens [2–5].

The genus *Pseudomonas* is known for its remarkable metabolic diversity, synthesizing a wide array of secondary metabolites with potent antimicrobial properties, which have resulted in several members of the genus being registered as BCAs against pathogens causing diseases in diverse crops [6–9]. Our previous work uncovered the antimicrobial activity of *Pseudomonas protegens* PBL3 against the rice pathogen *B. glumae*, the causal agent of bacterial panicle blight [10–14]. We also found that this antimicrobial activity was associated with molecules secreted by the bacteria (secretome) likely associated with putative secondary metabolites encoded by biosynthetic gene clusters (BGCs) [14]. To gain insight into the specific molecules in the *P. protegens* PBL3 secretome responsible for the antimicrobial activity, we tested eight of the genome-predicted compounds against *B. glumae* using commercially available analogues and found that only pyoverdine, pyoluteorin and 2,4-diacetylphloroglucinol (2,4-DAPG) inhibited the growth of *B. glumae* at high concentrations [15]. These molecules, however, did not fully explain the observed antimicrobial activity of the *P. protegens* PBL3 secretome; quantification of their endogenous concentrations by reversed-phase liquid chromatography with MS-MS, using the commercial analogues as standards, revealed that their endogenous concentrations were below the concentrations needed for activity [15]. The latter finding suggests that the antimicrobial activity of the *P. protegens* PBL3 secretome is either associated with the combination of predicted or uncharacterized secondary metabolites, encoded by previously identified BGCs, or by novel molecules encoded in other genomic regions.

The identification of naturally occurring antimicrobials is challenging due to their immense structural diversity, their production under specific growth/environmental conditions and technical limitations with bioanalytical approaches and compound identification [16–19]. Thus, an alternative approach that facilitates the identification of genomic regions associated with antimicrobial activity is comparative genomics [20–23].

Here, we applied a comparative genomics framework to identify genes and biosynthetic pathways associated with the antimicrobial activity of *P. protegens* PBL3 against *B. glumae*. To facilitate this approach, we selected six closely related *Pseudomonas* spp. strains with available genome sequences and differential antimicrobial activity against *B. glumae*. This differential antimicrobial activity led us to hypothesize that *Pseudomonas* spp. strains with antimicrobial activity against *B. glumae* have genomic regions in common with *P. protegens* PBL3 that are absent in *Pseudomonas* spp. strains without such antimicrobial activity. Our comparative genomic analysis among those *Pseudomonas* spp. strains and using the *P. protegens* PBL3 as a reference genome identified genomic regions uniquely present in the *P. protegens* PBL3 genome. This approach resulted in the identification of 188 annotated non-redundant putative genes. These genes span biosynthetic enzymes, metabolism, transporters, secretion systems and regulatory components, suggesting that the antimicrobial activity in *P. protegens* PBL3 is related not only to antimicrobial biosynthetic pathways but also to an integrated, multi-functional genetic network combining metabolite production, regulation and efficient export mechanisms.

## Methods

### Bacterial strains

Bacterial strains used in this study are listed in Table 1. *B. glumae* UAPB13 was grown in King’s B (KB) medium [24], and all the *Pseudomonas* strains were grown on Luria–Bertani medium [25]. Genomes of the *Pseudomonas* strains were obtained from NCBI and JGI Integrated Microbial Genomes and Microbiomes (IMG/M) [26].

**Table 1.** Bacterial strains used in the study

Strains	Source	Genome accession/IMG Genome ID
*Burkholderia glumae* UAPB13	Rice panicle	PRJNA702519; GCA_024611525.1
*Pseudomonas protegens* PBL3	Soil sample from wheat fields	PRJNA626017; GCA_016903635.1
*Pseudomonas protegens* 3295	Soil sample from sweet sorghum fields	2818991464
*Pseudomonas fluorescens* 513	Roots from pearl forage millet	2834028612
*Pseudomonas fluorescens* 1550	Rhizosphere from grain sorghum fields	2923586266
*Pseudomonas fluorescens* 4488	Rhizosphere from grain sorghum fields	2904518522
*Pseudomonas fluorescens* 1098	Rhizosphere from energy sorghum	2919063839
*Pseudomonas putida* 4512	Roots from false purple brome	2919228037
*P. protegens* CHA0	na	PRJNA78307; GCA_000397205.1

NCBI genome assemblies are identified by their GenBank accession numbers (GCA_). Genomes obtained from JGI IMG/M are identified by their IMG Genome ID (also referred to as Taxon ID). BioProject accessions are provided where available; they were not retrievable for all JGI IMG/M entries.

NA, not applicable.

### Secretome preparation and *in vitro* antimicrobial assay

Cell-free secretions (secretomes) were harvested from *P. protegens* PBL3, *P. fluorescens* 513, *P. fluorescens* 1550, *P. protegens* 3295, *P. fluorescens* 1098, *P. fluorescens* 4488 and *P. putida* 4512 for an antimicrobial assay against *B. glumae*, following previously described procedures [15]. To identify an appropriate time point for antimicrobial evaluation, the growth of *B. glumae*, measured as OD at 600 nm (OD_600_), was evaluated in the presence and absence of secretomes from the different *Pseudomonas* spp. strains. A single time point (18 h) was chosen as optimal, as it reflects differences among the strains. Secretomes were prepared from three biological replicates, and the antimicrobial assays were repeated three times, each time including three biological replicates and three technical replicates. Means and standard deviation for all treatment groups were calculated using all nine data points. Data were analysed with RStudio version 4.2.1. using Welch’s one-way ANOVA, with significance set at *P*<0.05. The Games–Howell significant difference test was used for post-hoc pairwise comparisons among treatment groups.

### Genome quality assessment and improvement

The quality of genomes was evaluated using QUAST (Quality Assessment Tool for Genome Assemblies) v 5.0.2 [27]. The draft genomes of the six *Pseudomonas* strains (*P. fluorescens* 513, *P. fluorescens* 1550, *P. protegens* 3295, *P. fluorescens* 1098, *P. fluorescens* 4488 and *P. putida* 4512) were enhanced by RagTag v2.1.0 [28] using *P. protegens* CHA0 and *P. protegens* PBL3 genomes as references to order and orient assembled contigs into longer sequences (scaffolds), by inserting gaps where the sequence was unknown.

### Identification and comparison of BGCs across *Pseudomonas* spp. strains

BGCs were predicted using Antibiotics and Secondary Metabolite Analysis Shell (antiSMASH) v 8.0.4 [29] for all seven *Pseudomonas* genomes analysed in this study. Each genome was processed with default parameters, enabling detection of secondary metabolite classes. BGC annotations generated for each strain were compiled into a presence–absence matrix to facilitate cross-strain comparison in reference to *P. protegens* PBL3.

### Comparative genomic analysis

Comparative genomic analyses were performed using complementary pangenome and whole-genome alignment approaches to identify genomic regions associated with antimicrobial activity. Comparative pangenomic analyses were performed using anvi’o (an analysis and visualization platform for omics data) v 8.0 [30] to estimate relationships among the genomes based on gene clusters — defined here by amino acid sequence homology across all analysed genomes, and distinct from the BGCs discussed elsewhere [31]. For the pangenomics workflow, annotated contigs-dbs were first created for the seven genomes and then used to generate a genomes-storage-db. This genome storage database was then used to run the pangenomic analysis using the command anvi-pan-genome. The contigs-dbs were annotated with the NCBI’s 2020 release of COGs (COG20) and the Kyoto Encyclopedia of Genes and Genomes (KEGG) databases v2023-09-22. Genomic regions of interest were defined as those present in the genomes of bacterial strains with antimicrobial activity and absent in the genomes of bacterial strains without antimicrobial activity. To extract these regions, a matrix of every gene cluster and its frequency of occurrence in each genome was obtained from the pangenomic profile [32].

To complement the pangenomic analysis and assess large-scale genome organization and synteny, whole-genome alignments were performed using progressiveMauve v 2.4.1 with default parameters [33, 34] to identify conserved, orthologous regions among all or subsets of the input genomes that are free from genome rearrangements (locally collinear blocks, LCBs). The backbone file generated by progressiveMauve, which contains genomic regions conserved across subsets of the analysed genomes, was used for downstream analyses. For each region, we recorded the number of antimicrobial-positive and -negative genomes that aligned to it. Regions present in all antimicrobial-positive strains and absent from all antimicrobial-negative strains were then retained for further analysis as candidate regions potentially associated with antimicrobial activity. Using the *P. protegens* PBL3 gene model as the basis, LCBs overlapping with annotated genes were aggregated to the gene level. If the start or end coordinate of an LCB fell into a known gene, it was assigned to the gene. In cases where multiple LCBs were assigned to the same gene, the minimum and maximum coordinates of these LCBs became the start and end coordinates, respectively, for the gene-level loci. Annotations were added to the resulting file using the GTF file of *P. protegens* PBL3 obtained from NCBI and supplemented by KEGG functional annotations from anvi’o.

The resulting candidate genes were visualized in Integrative Genomics Viewer (IGV) to examine their physical distribution across the *P. protegens* PBL3 genome. Genes were grouped into genomic clusters programmatically, when adjacent genes were separated by ≤1 kb. This threshold was selected as a conservative operational criterion based on intergenic distance distributions within experimentally validated *P. protegens* biosynthetic clusters and was found to be robust across a range of alternative cutoffs (500 bp to 2 kb). Putative operons within these clusters were inferred using operon predictions from the PathoLogic program in Pathway Tools v29.0, rather than by manual assignment [35]. Cluster-level functional interpretation as cluster module was then based on gene annotation, and the presence of shared biological themes among neighbouring genes within each cluster.

### Phylogenetic analysis and average nucleotide identity

The phylogenomic analysis on *P. protegens* PBL3 and the six genomes followed the ‘Pangenomic+Phylogenomics’ workflow [31]. Single-copy core gene clusters were extracted from the pangenomics profile and used in the phylogenomic analysis. Anvi’o’s anvi-gen-phylogenomic-tree utilizes FastTree [36] by default to generate a phylogenomic tree. Average nucleotide identity (ANI) for the seven genomes was computed using the anvi’o command anvi-compute-genome-similarity [37, 38].

## Results and discussions

### Genetically related *Pseudomonas* spp. strains exhibit differential antimicrobial activity against *B. glumae*

To facilitate comparative genomic analysis for gene mining in *P. protegens* PBL3, we necessitated the identification of closely related *Pseudomonas* spp. strains with fully sequenced genomes and contrasting phenotypes. For that purpose, we obtained six *Pseudomonas* spp. strains: *P. fluorescens* 513, *P. fluorescens* 1550, *P. protegens* 3295, *P. fluorescens* 1098, *P. fluorescens* 4488 and *P. putida* 4512 and extracted their respective secretomes to evaluate their antimicrobial activity against *B. glumae* and compare it with the antimicrobial activity of *P. protegens* PBL3.

To characterize the antimicrobial activity of the different *Pseudomonas* spp. strains, we evaluated the effect of their secretomes on *B. glumae* growth (Fig. S1, available in the online Supplementary Material). *B. glumae* grown in KB medium alone (control) began exponential phase at 6 h and reached an OD_600_ of ~2.5 by 21 h. Secretomes from *P. fluorescens* 1098, *P. fluorescens* 4488 and *P. putida* 4512 showed growth patterns similar to the control condition. In contrast, secretomes from *P. protegens* PBL3, *P. fluorescens* 513, *P. fluorescens* 1550 and *P. protegens* 3295 did not start exponential phase, and the OD_600_ values remained below 0.5 throughout all the time points. Because these distinct response patterns were already clearly separated by 18 h, this time point was selected for three additional assays, including all biological and technical replications.

**Fig. 1.**
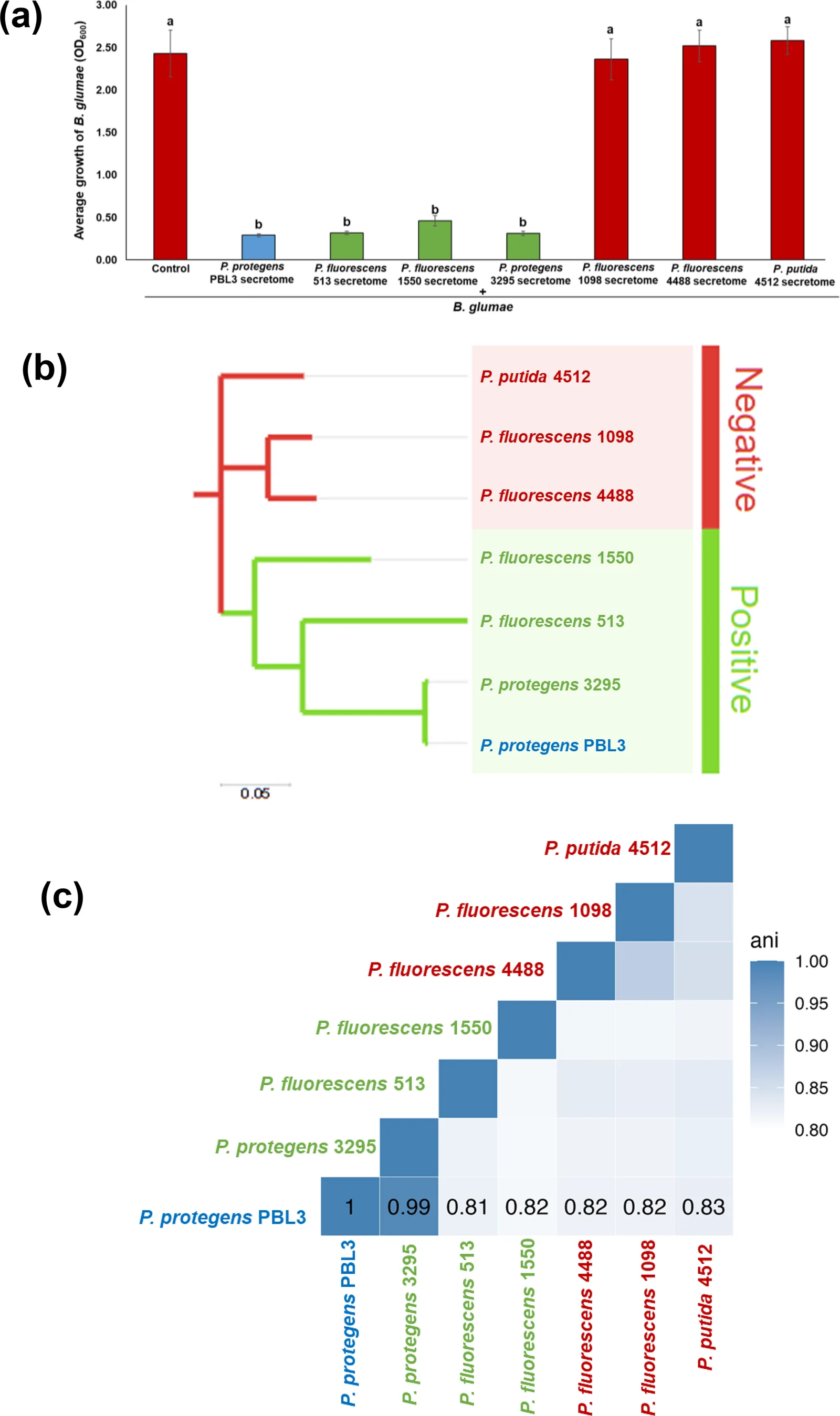
Antimicrobial activity of different strains of *Pseudomonas* against *B. glumae* and their genomic relatedness. (**a**) *B. glumae* at an OD_600_ of 0.2 was diluted in KB broth and mixed with water (control), and secretome from different strains of *P. protegens* PBL3 (treatments). The growth of *B. glumae* was evaluated after 18 h. *Pseudomonas* spp. strains lacking antimicrobial activity are shown in red, *Pseudomonas* spp. strains with antimicrobial activity in green and *P. protegens* PBL3 in blue. Bars represent the average growth of *B. glumae*, and error bars indicate standard deviation calculated from nine observations generated from three independent biological replicates, each with three technical replicates. Statistical significance indicated by letters above the bars was determined using a Welch’s one-way analysis of variance with the Games–Howell difference test (*P*-value <0.05). (**b**) Phylogenomic tree of seven *Pseudomonas* strains inferred from single-copy core genes using anvi’o. Branch lengths are proportional to the number of substitutions per site, as indicated by the scale bar. (**c**) Pairwise ANI heatmap showing genomic relatedness of *P. protegens* PBL3 with the other *Pseudomonas* strains, calculated using anvi’o. Colour intensity reflects ANI values, with darker blue indicating higher nucleotide identity. Bacterial names in panels (**b**) and (**c**) are colour-coded based on antimicrobial phenotypes (**a**) .

At 18 h, *B. glumae* grown in KB medium alone (control) reached an OD_600_ of 2.5, whereas the growth of *B. glumae* in KB supplemented with the *P. protegens* PBL3 secretome was reduced by 88% as previously reported [15]. Among the six additional *Pseudomonas* strains tested, the secretomes of *P. fluorescens* 513, *P. fluorescens* 1550 and *P. protegens* 3295 exhibited antimicrobial activity against *B. glumae* similar to the antimicrobial activity observed with *P. protegens* PBL3, causing a reduction in *B. glumae* growth by 87, 81 and 87%, respectively. In contrast, the secretomes of *P. fluorescens* 1098, *P. fluorescens* 4488 and *P. putida* 4512 had no antimicrobial activity against *B. glumae*, and the levels of *B. glumae* growth were equivalent to those under control conditions (Fig. 1a, Table S1). Thus, *P. fluorescens* 513, *P. fluorescens* 1550 and *P. protegens* 3295 were considered as *B. glumae* antimicrobial-positive strains, whereas *P. fluorescens* 1098, *P. fluorescens* 4488 and *P. putida* 4512 were considered as *B. glumae* antimicrobial-negative strains.

To assess whether the antimicrobial activity is linked to the evolutionary relationship among strains having antimicrobial activity, we generated a phylogenomic tree and found that the three strains with antimicrobial activity against *B. glumae* (*B. glumae* antimicrobial-positive strains), *P. protegens* 3295, *P. fluorescens* 513 and *P. fluorescens* 1550, are in the same cluster with *P. protegens* PBL3, whereas the strains lacking antimicrobial activity against *B. glumae* (*B. glumae* antimicrobial-negative strains), *P. fluorescens* 4488, *P. fluorescens* 1098 and *P. putida* 4512, were in a different cluster (Fig. 1b), indicating that the antimicrobial activity is partly associated with the shared evolutionary history among strains. However, when we assessed genome relatedness between *P. protegens* PBL3 and the other strains using ANI, we found 99% identity between *P. protegens* PBL3 and *P. protegens* 3295, but that percentage of identity is reduced to ~81–83% with other *Pseudomonas* spp., including strains without antimicrobial activity but also *P. fluorescens* 513 and *P. fluorescens* 1550 that have antimicrobial activity (Fig. 1c), highlighting the potential to dissociate phylogenetic relatedness from antimicrobial phenotypes.

### *Pseudomonas* strains harbour distinct, strain-specific repertoires of BGCs

Before starting genome comparisons, we curated the draft genomes of *Pseudomonas* strains *P. fluorescens* 513, *P. fluorescens* 1550, *P. protegens* 3295, *P. fluorescens* 1098, *P. fluorescens* 4488 and *P. putida* 4512 by scaffolding using the *P. protegens* CHA0 and *P. protegens* PBL3 as reference genomes. We found that all six draft genomes were at least 99% identical in assembly contiguity regardless of the reference genome used (Table S2). Scaffolding, using reference genomes, reduced the number of contigs while increasing the length of contigs, as demonstrated by higher N50 values and obtaining an L50 of one for all of the six *Pseudomonas* strains (Table S2). L50 of one indicated that a single contig was sufficient to cover half of the genome assembly. This curation provided fewer, longer contigs, resulting in a more complete and less fragmented genome assembly. Because the resulting reordered assemblies were highly similar between the two reference genomes, we used *P. protegens* PBL3 for downstream analyses as it is our genome of interest in this study.

**Fig. 2.**
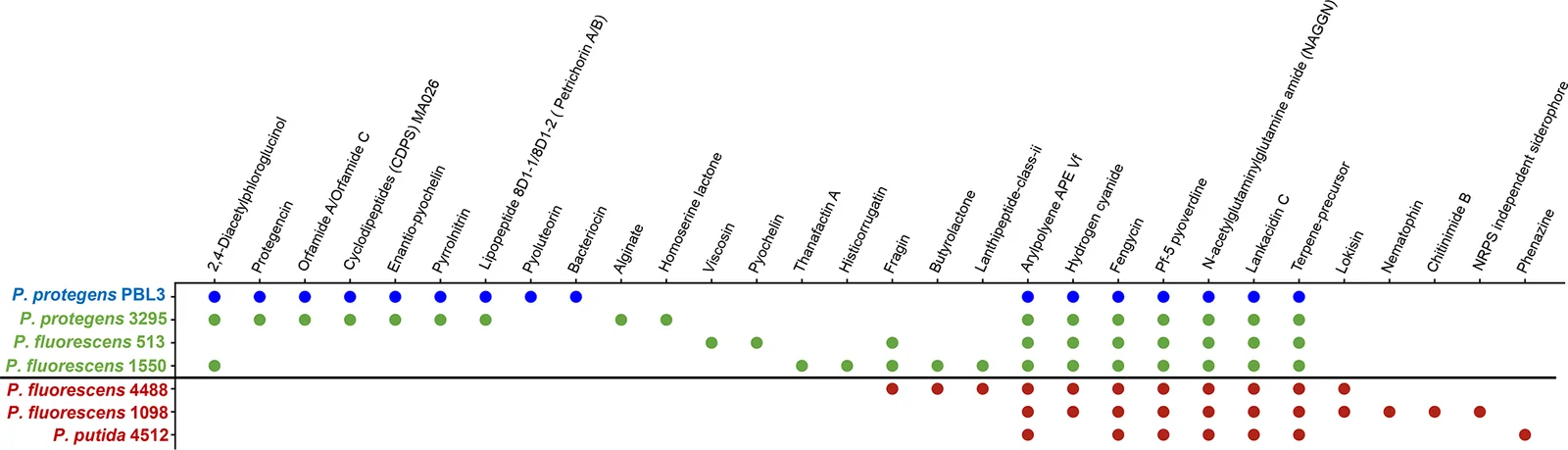
Distribution of predicted BGCs across *Pseudomonas* strains with differential antimicrobial activity against *B. glumae*. Presence–absence matrix of BGC predicted using antiSMASH v8.0.4 across seven *Pseudomonas* spp. genomes. Each column represents a predicted BGC, annotated by the putative metabolite class or product, and each row represents an individual strain (coloured by phenotype: *P. protegens* PBL3 in blue, antimicrobial-positive in green and antimicrobial-negative in red). Coloured circles indicate the presence of a given BGC in a strain. The horizontal line separates antimicrobial-positive and antimicrobial-negative strains.

We previously found that *P. protegens* PBL3 encoded 12 distinct BGCs predicted to encode secondary metabolites [14]. To gain insight into the coding capacity of the additional six *Pseudomonas* spp. with differential antimicrobial activity against *B. glumae*, we compared their BGC profiles against the *P. protegens* PBL3 BGC profile using antiSMASH v8.0.4. (Fig. 2). This analysis revealed *P. protegens* PBL3 is predicted to encode four new BGCs: protegencin, hydrogen cyanide, lankacidin C and terpene precursor. Comparative analysis of BGCs across all *Pseudomonas* strains with and without antimicrobial activity against *B. glumae* identified six BGCs predicted to encode arylpolyene APE Vf, fengycin, Pf-5 pyoverdine, *N*-acetylglutaminylglutamine amide, lankacidin C and a terpene precursor; the BGC predicted to encode hydrogen cyanide was present in six of the strains and absent in *P. putida* 4512. When comparing *B. glumae* antimicrobial-positive strains, *P. protegens* 3295 and *P. protegens* PBL3, which have the closest genetic relationship, have seven common BGCs: 2,4-DAPG, protegencin, orfamide A/orfamide C, cyclodipeptides, enantio-pyochelin, pyrrolnitrin and lipopeptide 8D1-1/8D1-2 (Petrichorin A/B). In contrast to *P. protegens* PBL3, *P. protegens* 3295 lacks pyoluteorin and bacteriocin BGCs and includes two additional BGCs predicted to encode alginate and homoserine lactone. The additional *B. glumae* antimicrobial-positive strains within the *P. fluorescens* group have genomes that predict specific BGCs, such as viscosin and pyochelin in *P. fluorescens* 513, and thanafactin A and histicorrugatin in *P. fluorescens* 1550; both *P. fluorescens* 513 and *P. fluorescens* 1550 genomes contain BGCs predicted to encode fragin, which is also encoded by the *B. glumae* antimicrobial-negative strain, *P. fluorescens* 4488. Additionally, *P. fluorescens* 1550 encoded 2,4-DAPG, which is shared with both *P. protegens* strains, as well as butyrolactone and lanthipeptide-class-ii cluster, which were also present in the *B. glumae* antimicrobial-negative strain, *P. fluorescens* 4488. Further, *Pseudomonas* spp. strains lacking antimicrobial activity against *B. glumae* encoded additional BGCs: lokisin in *P. fluorescens* 4488 and *P. fluorescens* 1098; nematophin, chitinimide B and a non-ribosomal peptide synthetase (NRPS)-independent siderophore in *P. fluorescens* 1098; and a phenazine BGC in *P. putida* 4512.

Overall, this analysis revealed that *Pseudomonas* spp. genomes with antimicrobial activity against *B. glumae* are endowed with more BGCs than those without it. Importantly, it appears that this antimicrobial activity is associated with combinations of BGCs rather than specific BGCs, as we initially hypothesized [15]. Moreover, the antiSMASH results also revealed that predicted BGCs are strain specific, further dissociating phylogenetic relationships from antimicrobial activities.

Although these results are insightful, this analysis using antiSMASH did not allow us to define the specific BGCs associated with the antimicrobial activity of *P. protegens* PBL3. This may be due to antiSMASH limitations regarding its dependency on existing rule-based detection, predicting canonical BGCs and not capturing non-BGC genes or divergent biosynthetic pathways, such as regulatory, transport, secretion or accessory metabolic genes that may contribute to antimicrobial activity [39].

### Comparative genomics identifies genomic regions uniquely associated with antimicrobial activity in *P. protegens* PBL3

**Fig. 3.**
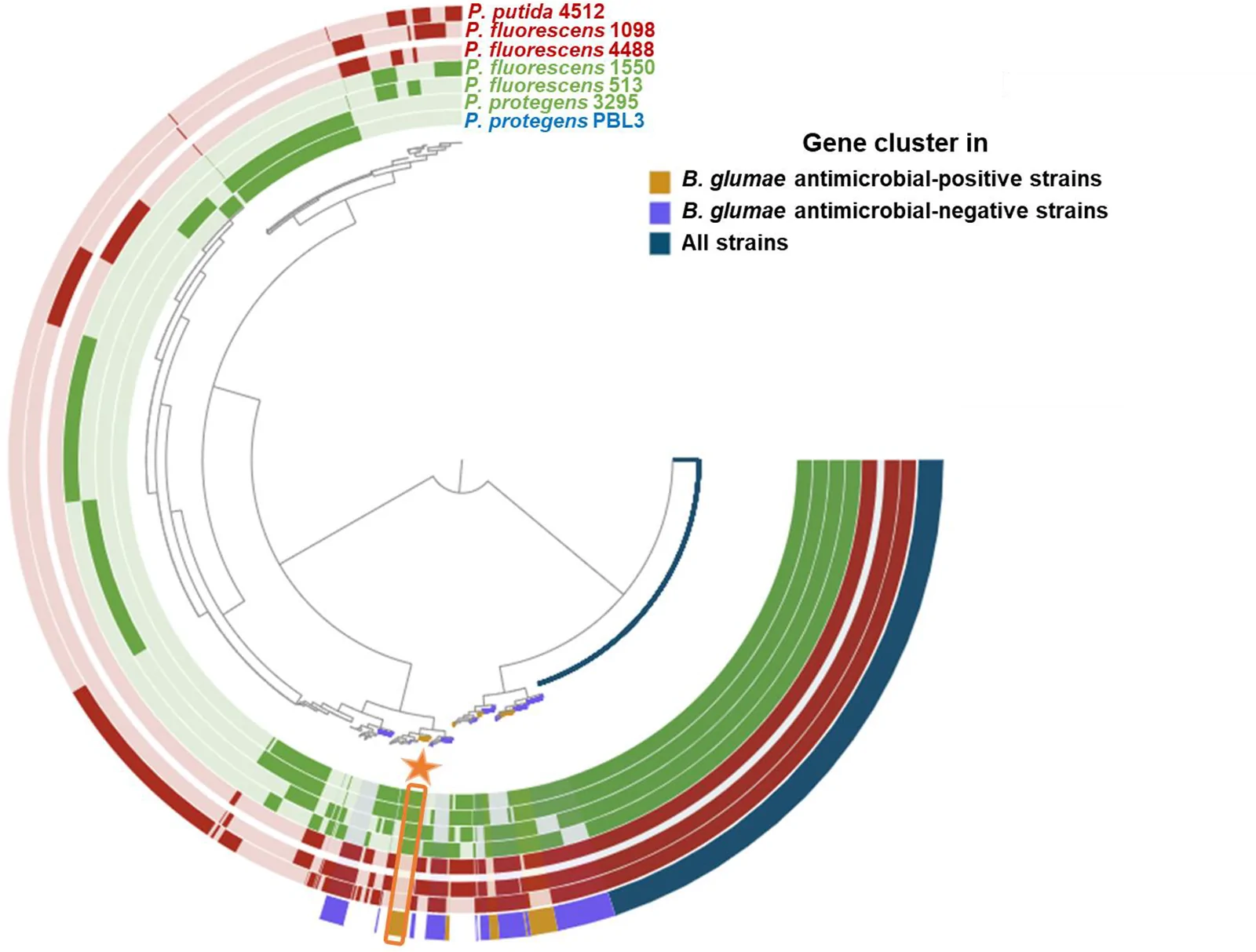
Comparative genomics identifies genes uniquely associated with antimicrobial activity against *B. glumae*. Circular pangenome visualization of seven *Pseudomonas* genomes generated using the anvi’o pangenomics workflow. The circular dendrogram was constructed based on the presence or absence of gene clusters (orthologous gene families) across genomes. Concentric rings represent individual genomes for *B. glumae* antimicrobial-positive strains in green and *B. glumae* antimicrobial-negative strains in red; coloured blocks indicate the presence of gene clusters in that genome. The outermost ring summarizes gene cluster distribution patterns across phenotypic groups: gold blocks indicate gene clusters exclusive to antimicrobial-positive strains, purple blocks indicate gene clusters exclusive to antimicrobial-negative strains and dark blue blocks denote gene clusters present in both antimicrobial-positive and antimicrobial-negative strains. The gold rectangle with star highlights a genomic region enriched in gene clusters uniquely conserved among antimicrobial-positive strains but absent from antimicrobial-negative strains.

The comparative BGC profiling analysis using antiSMASH challenged our hypothesis regarding the conservation of BGCs among strains with activity and the absence of those BGCs in the strains without activity. Thus, we opted to generate more robust information on genomic regions associated with antimicrobial activity by comparative genomics. We initially compared all seven genomes at the amino acid level using anvi’o to identify core gene clusters defined as an orthologous group of homologous genes identified across genomes. The resulting pangenome comprised 40,762 genes, of which 22,518 genes (55.0%) constituted the core genome (Fig. 3, dark blue band). Within phenotypic groups, strains with antimicrobial activity (*B. glumae* antimicrobial-positive) (including *P. protegens* PBL3) shared 1,684 genes (310 clusters) (Fig. 3, gold band), while strains lacking antimicrobial activity (*B. glumae* antimicrobial-negative) shared 4,008 genes (778 clusters) (Fig. 3, purple band). To identify genomic regions potentially associated with antimicrobial activity, we focused on genes within the clusters present in all *B. glumae* antimicrobial-positive strains and *P. protegens* PBL3 but absent in all *B. glumae* antimicrobial-negative strains (Fig. 3, gold rectangle with star). This analysis revealed 98 genes (96 clusters) unique to strains with antimicrobial activity, as genomic differences associated with antimicrobial activity (Fig. 3, Table S3). When the anvi’o pangenomic analysis was repeated using unscaffolded assemblies, all 98 candidate genes were independently recovered, indicating that reference-guided scaffolding did not substantially affect gene presence/absence patterns in the pangenomic analysis. These identified 98 genes represent potential candidates for functional characterization.

Since anvi’o pan-genome analysis does not capture subtle differences at the nucleotide level, such as small insertions, deletions or point mutations, we complemented the anvi’o analysis with a whole-genome multiple sequence alignment using progressiveMauve to generate more robust information. progressiveMauve identified 223 unique genomic hits that are present in *P. protegens* PBL3 and in other strains showing antimicrobial activity (*B. glumae* antimicrobial-positive strains) but absent in strains lacking antimicrobial activity (*B. glumae* antimicrobial-negative strains) (Table S4). Out of these, 161 correspond to annotated genes, while 62 regions are uncharacterized. The uncharacterized regions suggested that genes encode novel mechanisms that contribute to antimicrobial activities.

Together, these two complementary approaches resulted in 188 annotated non-redundant putative genes potentially associated with antimicrobial activity, 27 genes unique to anvi’o and 87 genes unique to progressiveMauve, with 74 genes overlapping between the 2 analyses (Table S5).

### Antimicrobial-associated genes localize within predicted BGCs in *P. protegens* PBL3

**Fig. 4.**
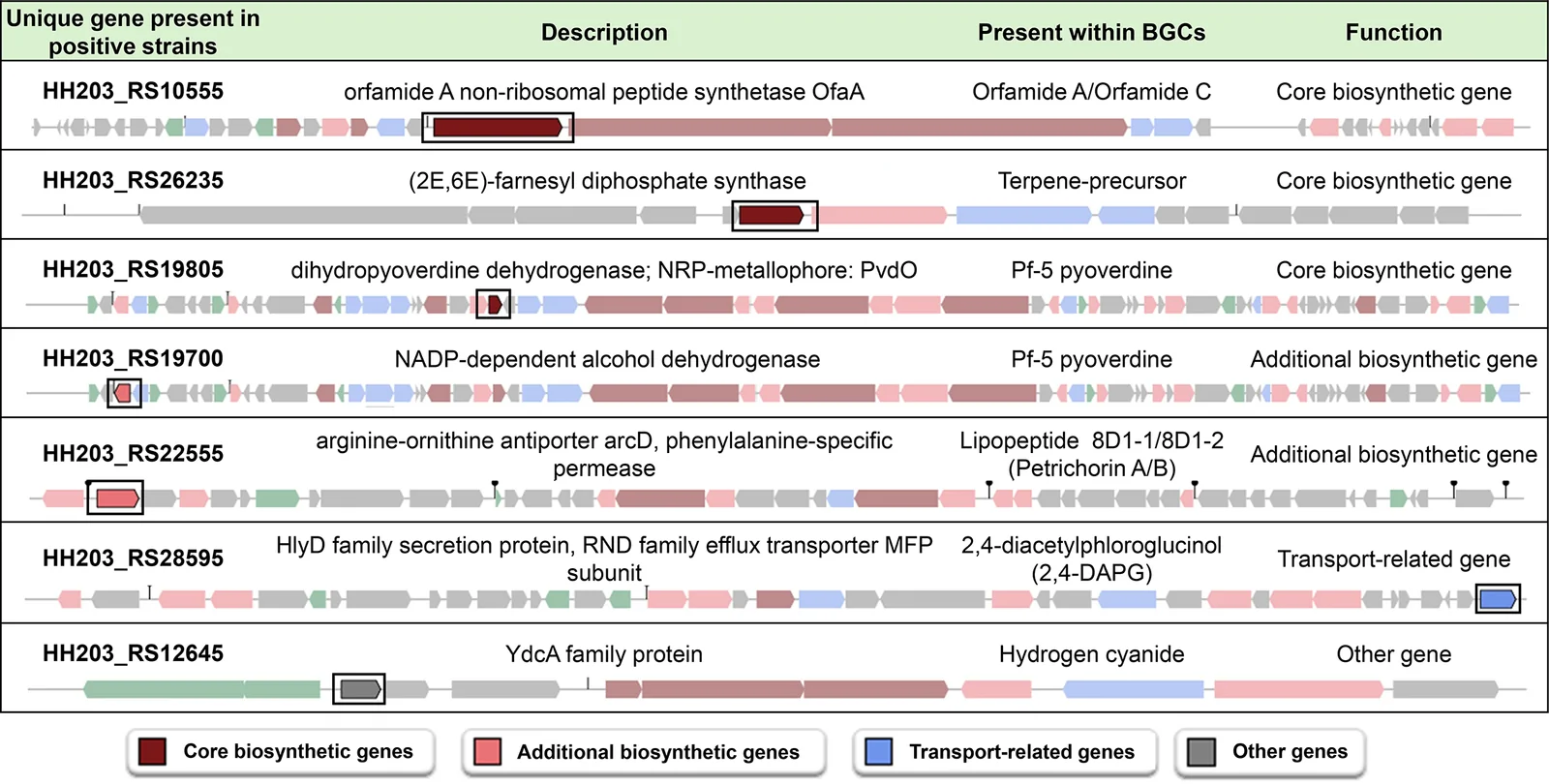
Antimicrobial-associated genes mapped to predicted BGCs in *P. protegens* PBL3. Linear genomic representations of BGCs: orfamide A/C, terpene precursor, Pf-5 pyoverdine, lipopeptide 8D1-1/8D1-2 (Petrichorin A/B), 2,4-DAPG and HCN in the *P. protegens* PBL3 genome predicted by antiSMASH. Each block arrow represents a gene and its orientation within the cluster. Genes uniquely conserved among antimicrobial-positive strains are highlighted with black outlines. Genes are colour-coded by functional category: core biosynthetic genes (maroon), additional biosynthetic genes (light red), transport-related genes (blue) and other genes (grey).

To determine whether any of the 188 genes uniquely present in *P. protegens* PBL3 and the *B. glumae* antimicrobial-positive strains were directly associated with known secondary metabolite biosynthesis, we examined their genomic locations relative to the BGCs predicted in *P. protegens* PBL3 using antiSMASH (Fig. 2). This mapping revealed that 7 of the 188 candidate genes fell within 6 BGCs previously identified (Fig. 4). Genes HH203_RS10555, HH203_RS26235 and HH203_RS19805 mapped to orfamide A/orfamide C cluster, terpene precursor cluster and pyoverdine cluster, respectively, and encode core biosynthetic genes. Two additional genes, HH203_RS19700 mapped to the pyoverdine cluster and HH203_RS22555 mapped to the lipopeptide 8D1-1/8D1-2 (Petrichorin A/B) cluster, are associated with additional biosynthetic functions. Within the 2,4-DAPG cluster, gene HH203_28595 encodes an RND-family efflux transporter, indicating that antimicrobial activity may also depend on metabolite export associated with BGC function. The remaining candidate, HH203_RS12645 in the hydrogen cyanide cluster, encodes a YdcA family protein, whose function remains poorly characterized. Although we had already identified these clusters with the *P. protegens* PBL3 genomic sequencing, these results highlight specific genes within those clusters as uniquely present in *B. glumae* antimicrobial-positive strains, suggesting that those genes are essential for the production of a specific metabolite different from the commercial standards used in previous analyses [15]. This pattern is consistent with biosynthetic processes mediated by the activities of NRPSs, such as the synthesis of orfamide, pyoverdine and lipopeptides, and polyketide synthases, such as the synthesis of 2,4-DAPG, whose activities can generate a variety of structurally diverse molecules without changes at the genomic level [8, 40–42].

### KEGG-based functional categorization reveals a multi-functional network of antimicrobial-associated genes in *P. protegens* PBL3

**Fig. 5.**
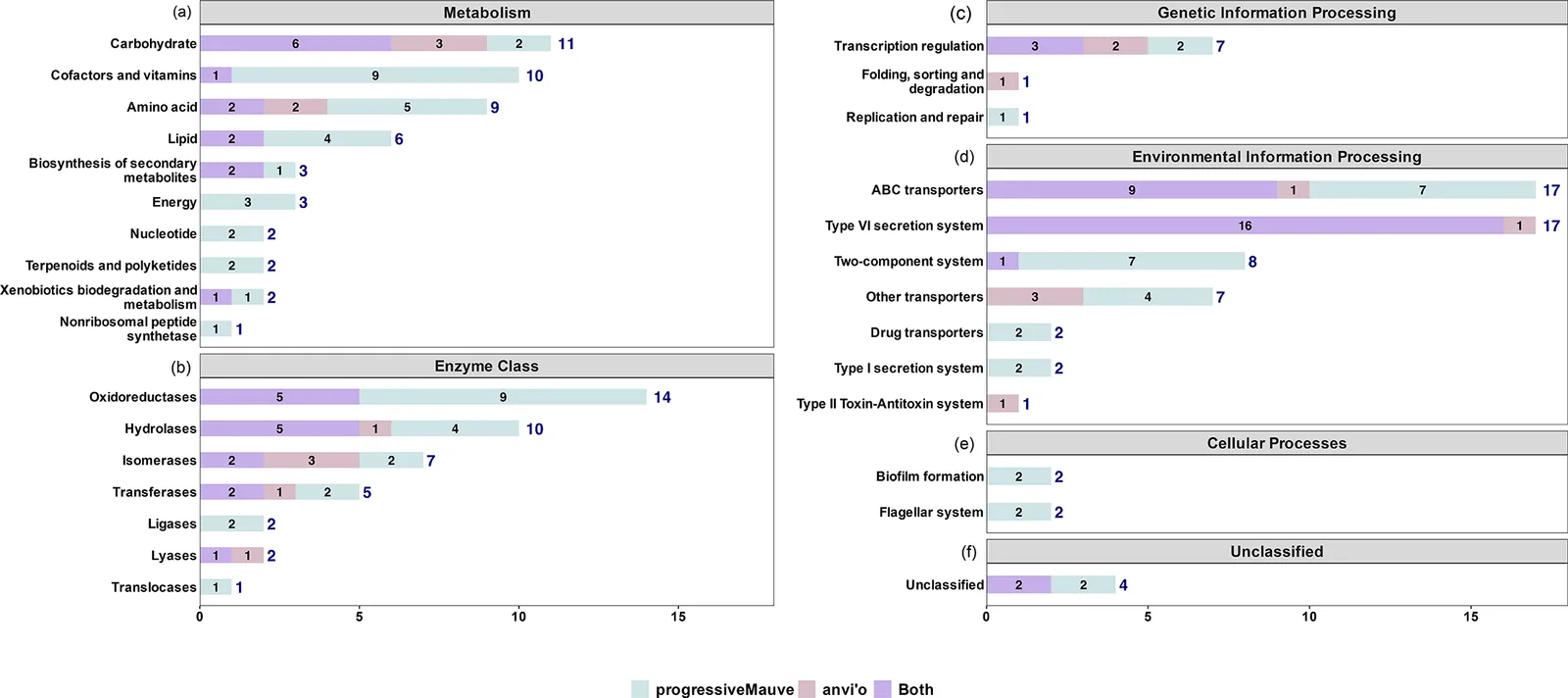
KEGG-based functional classification of genes uniquely associated with antimicrobial activity in *P. protegens* PBL3. Stacked bar plots summarizing KEGG functional assignments for genes uniquely present in antimicrobial-positive strains (including *P. protegens* PBL3) and absent in antimicrobial-negative strains. Genes were annotated using KO assignments and grouped into major functional categories indicated in the panel as (**a**) metabolism, (**b**) enzyme class, (**c**) genetic information processing, (**d**) environmental information processing, (**e**) cellular processes and (**f**) unclassified. Bars represent the number of genes assigned to each group within the major functional category, with colour coding indicating the method by which genes were identified: progressiveMauve only (light blue), anvi’o only (light brown) or overlapping between both methods (light purple). Numbers at the end of each bar indicate total gene counts per functional group.

The 7 genes that are located in previously known metabolite-producing clusters represent only a small fraction of the 188 candidate genes identified through comparative genomics. This suggests that genes within the canonical BGC profiling alone were insufficient to explain the antimicrobial phenotype in *P. protegens* PBL3 and that antimicrobial activity-associated genes may extend well beyond classical secondary metabolite pathways. Instead, comparative genomics identified both BGC-linked and non-BGC-linked candidate genes that may collectively contribute to antimicrobial activity. To better understand the potential biological roles of these candidate genes, we performed KEGG-based functional annotation of all 188 genes uniquely present in *P. protegens* PBL3. KEGG Orthology (KO) accessions assigned by anvi’o were mapped to the KEGG Pathway and BRITE databases, followed by manual curation, where necessary, to generate functionally meaningful groupings. This analysis enabled us to categorize the genes into major functional classes, including metabolism, enzyme class, genetic information processing, environmental information processing and cellular processes (Fig. 5, Table S6), providing a broader view of the molecular functions potentially contributing to antimicrobial activity. To capture the complete repertoire of candidates, KEGG analysis was performed separately for high-confidence genes detected by both comparative methods (Fig. 5, light purple bars) and for those identified uniquely by either anvi’o (Fig. 5, light brown bars) or progressiveMauve (Fig. 5, light blue bars).

Because a single gene can function in multiple pathways, some genes were represented across both metabolic and enzyme class categories (Table S5). Within the metabolism network, genes were distributed across ten functional groups involved in the biosynthesis of structurally diverse antimicrobial metabolites. The largest representation was in carbohydrate metabolism, followed by metabolism of cofactors and vitamins, amino acid metabolism, lipid metabolism, biosynthesis of secondary metabolites, energy metabolism, nucleotide metabolism, terpenoid and polyketide metabolism, xenobiotic biodegradation and metabolism and NRPS metabolism (Fig. 5a). These metabolic pathways provide the essential precursors, biochemical intermediates and cofactors that support antimicrobial metabolite biosynthesis in *P. protegens* PBL3. Enzyme classification further resolved the repertoire of catalytic functions encoded by these genes. The distribution was dominated by oxidoreductases, followed by hydrolases, isomerases, transferases, ligases, lyases and translocases (Fig. 5b). Many of these enzymes mediate activation, modification and diversification reactions that enhance the structural complexity and bioactivity of antimicrobial compounds. Collectively, these non-exclusive groups define the biochemical versatility and broad metabolic reservoir likely required for the antimicrobial metabolite production in *P. protegens* PBL3.

Beyond metabolism and enzymatic functions, KEGG annotation revealed genes uniquely associated with genetic information processing, environmental information processing and cellular processes (Table S5), which collectively contribute to regulation, secretion, transport and stress adaptation associated with antimicrobial activity. A total of nine genes were classified as genetic information processing, including seven transcription regulation and one gene each for protein folding, sorting and degradation, and replication and repair (Fig. 5c). Fifty-four genes were classified as environmental information processing, encompassing diverse transport systems, secretion machinery and regulatory modules. The largest groups included ABC transporters and the type VI secretion system (T6SS), each with 17 genes. Eight genes encoded two-component systems (TCSs), seven genes corresponded to other transporters, two genes to drug transporters, two genes to the Type I secretion system and one gene to a Type II toxin–antitoxin system (Fig. 5d). Four genes were classified under cellular processes category, including two involved in biofilm formation and two in the flagellar system (Fig. 5e). Four genes lacked definitive pathway assignments and were categorized as unclassified (Fig. 5f).

Collectively, genes classified under genetic information processing, environmental information processing and cellular processes form an integrated regulatory and export network that complements core metabolic and enzymatic pathways. The KEGG-based functional classification thus highlights a complex, multi-functional network of candidate genes in antimicrobial-producing strains, encompassing biosynthetic enzymes, transporters, secretion systems and transcriptional regulators. These findings suggest that *P. protegens* PBL3 potentially employs not only classical biosynthetic pathways but also coordinated regulatory, metabolic and export modules to synthesize and deploy antimicrobial compounds.

### Identification of antimicrobial activity-associated gene clusters in *P. protegens* PBL3 genome uncovers multiple functional categories

**Fig. 6.**
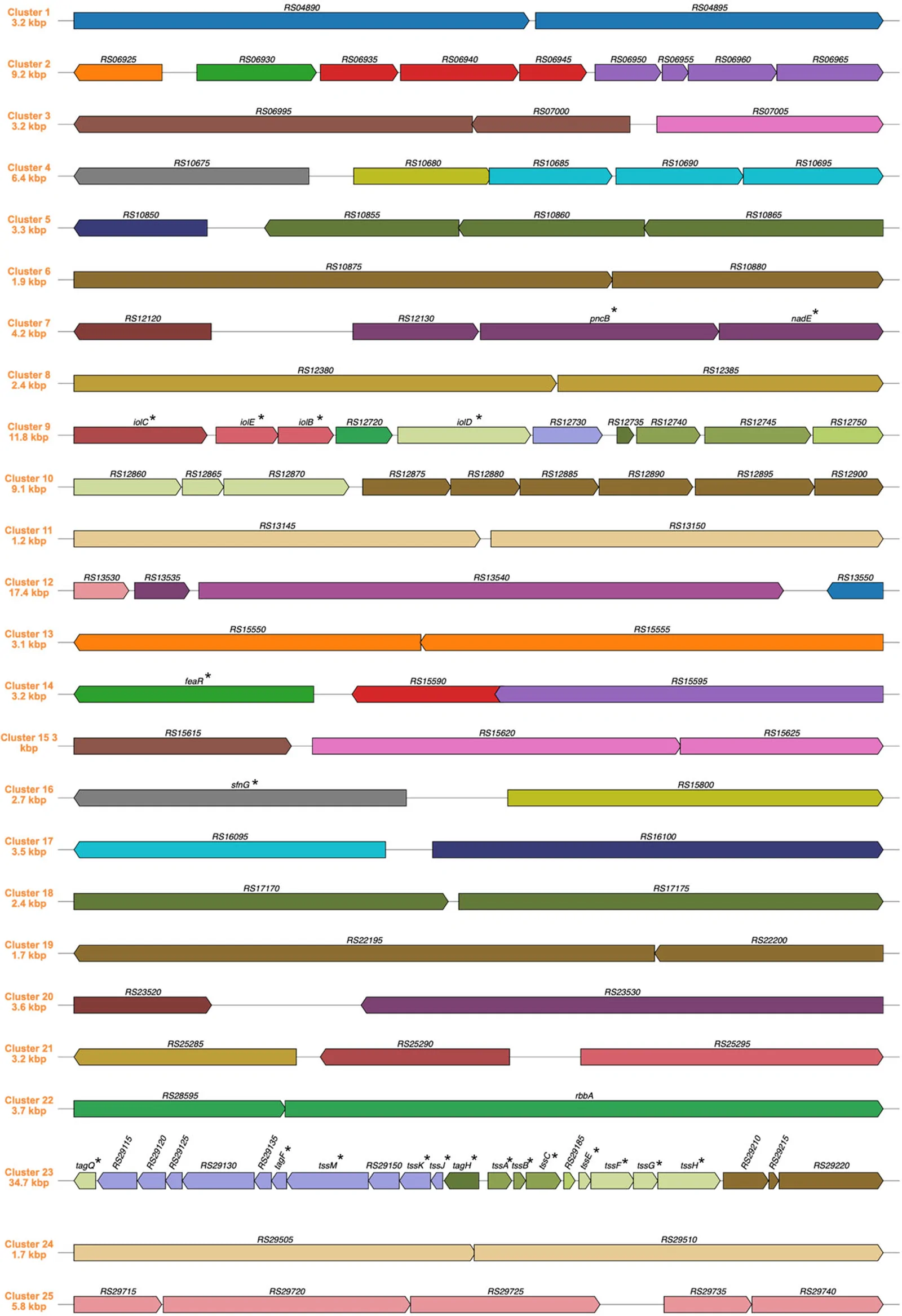
Genomic organization of antimicrobial-associated gene uniquely present in *P. protegens* PBL3. Linear maps of 25 discrete genomic clusters identified in *P. protegens* PBL3 that harbour genes uniquely present in antimicrobial-producing strains and absent in antimicrobial-negative strains. Clusters were defined as sets of adjacent genes separated by ≤1 kb and frequently containing one or more putative operons. Arrows represent predicted open reading frames oriented by transcriptional direction; genes sharing the same colour within a cluster indicate putative operons. Clusters are numbered (Clusters 1–25) according to their genomic position in the *P. protegens* PBL3 genome, with total cluster sizes indicated in kilobases (kbp).

To contextualize the 188 candidate genes uniquely present in *P. protegens* PBL3 and the antimicrobial-positive strains, we examined their physical distribution across the genome. Manual inspection using IGV revealed that these genes were not randomly distributed but formed 25 discrete genomic clusters ranging in size from 1.2 to 34.7 kb that were numbered based on their genomic position in *P. protegens* PBL3 (Fig. 6). Each of these genomic clusters comprises sets of adjacent genes encompassing 2 to 23 genes separated by no more than 1 kb and frequently containing 1 or more putative operons. Because specialized metabolic functions and antimicrobial pathways in *Pseudomonas* are often encoded in operon-like loci, this architecture suggests that these regions may represent candidate antimicrobial-associated modules. Functional annotation of the genes based on gene description within each cluster exhibits a coherent biological theme, allowing us to infer higher-order functional modules for each cluster (Tables 2 and S7).

**Table 2.** Antimicrobial-associated genes in *P. protegens* PBL3 are organized into 25 putative functional clusters

Cluster	Gene ID/symbol*	Gene description	Proposed cluster module
1	HH203_RS04890	DUF1302 domain-containing protein	**Uncharacterized**
	HH203_RS04895	DUF1329 protein; TadD-like Flp pilus assembly protein	
2	HH203_RS06925	GntR family transcriptional regulator	**Cell wall remodelling, metabolism and polyamine-transport module**
	HH203_RS06930	Cell-wall amidase (GatA-like)	
	HH203_RS06935	Polysaccharide deacetylase family protein	
	HH203_RS06940	MFS family transporter (NarK-like)	
	HH203_RS06945	SDR family NAD(P)-dependent oxidoreductase	
	HH203_RS06950	SDR family NAD(P)-dependent oxidoreductase	
	HH203_RS06955	Hypothetical protein	
	HH203_RS06960	CobW family GTP-binding metallochaperone	
	HH203_RS06965	ABC spermidine/putrescine SBP PotD	
3	HH203_RS06995	Rhodanese-related sulfurtransferase	**Sulphur metabolism and transcriptional regulation module**
	HH203_RS07000	Cysteine dioxygenase/3-mercaptopropionate dioxygenase	
	HH203_RS07005	LysR family transcriptional regulator	
4	HH203_RS10675	Sigma-54-dependent Fis family regulator (AcoR-like)	**Acetoin/pyruvate dehydrogenase module**
	HH203_RS10680	NAD^+^ kinase	
	HH203_RS10685	TPP-dependent dehydrogenase E1 component alpha	
	HH203_RS10690	Alpha-ketoacid dehydrogenase E1 beta subunit	
	HH203_RS10695	Dihydrolipoyllysine acetyltransferase/hydrolase	
5	HH203_RS10850	PQQ-dependent catabolism-associated CXXCW motif protein	**Redox enzyme, multidrug ABC efflux module**
	HH203_RS10855	ABC-type multidrug transport permease YadH	
	HH203_RS10860	ABC transporter ATP-binding protein CcmA	
	HH203_RS10865	YVTN family *β*-propeller repeat protein YncE	
6	HH203_RS10875	HAMP-domain sensor histidine kinase (NarL family)	**Two-component regulatory module**
	HH203_RS10880	NarL/FixJ family response regulator	
7	HH203_RS12120	NUDIX hydrolase (ADP-ribose pyrophosphatase-like)	**NAD biosynthesis/salvage module**
	HH203_RS12130	Nicotinamidase PncA	
	pncB	Nicotinate phosphoribosyltransferase PncB	
	nadE	NAD^+^ synthase NadE	
8	HH203_RS12380	Type 2 ATP-grasp family protein	**Putative peptide enzymatic module**
	HH203_RS12385	Alpha-E domain-containing protein	
9	iolC	5-Dehydro-2-deoxygluconokinase/aldolase (IolC-like)	**Myo-inositol utilization and sugar ABC transport module**
	iolE	Myo-inosose-2 dehydratase	
	iolB	5-Deoxy-d-glucuronate isomerase IolB	
	HH203_RS12720/ioll	TIM-barrel sugar epimerase/2-keto-myo-inositol isomerase	
	iolD	THcHDO acylhydrolase IolD	
	HH203_RS12730	Myo-inositol dehydrogenase (MviM-like)	
	HH203_RS12735	Hypothetical protein	
	HH203_RS12740	Sugar ABC transporter SBP (RbsB-like)	
	HH203_RS12745	Sugar ABC transporter ATPase (MglA-like)	
	HH203_RS12750	Sugar ABC transporter permease (AraH-like)	
10	HH203_RS12860	SfnB family sulphur acquisition oxidoreductase	**Branched-chain amino acid transport with redox metabolism module**
	HH203_RS12865	Acyl-CoA dehydrogenase	
	HH203_RS12870	LLM flavin-dependent monooxygenase (long-chain alkane monooxygenase)	
	HH203_RS12875	AMP-binding long-chain acyl-CoA synthetase	
	HH203_RS12880	ABC branched-chain amino acid transporter ATPase LivG	
	HH203_RS12885	ABC branched-chain amino acid permease LivH	
	HH203_RS12890	ABC branched-chain amino acid permease LivM	
	HH203_RS12895	ABC branched-chain amino acid SBP LivK	
	HH203_RS12900	ABC branched-chain amino acid transporter ATPase LivF	
11	HH203_RS13145	Hypothetical protein	**Uncharacterized**
	HH203_RS13150	Hypothetical protein	
12	HH203_RS13530	PstS-like phosphate ABC SBP	**Phosphate acquisition and multidrug efflux module**
	HH203_RS13535	PstS-like phosphate ABC SBP	
	HH203_RS13540	Filamentous haemagglutinin TamB-like protein	
	HH203_RS13550	MFS transporter AraJ (multidrug efflux)	
13	HH203_RS15550	HlyD family Type I secretion adaptor	**Type I secretion/efflux module**
	HH203_RS15555	Type I secretion permease/ATPase	
14	feaR	AraC family transcriptional regulator FeaR	**Regulated amino acid transport module**
	HH203_RS15590	DUF3156 family protein	
	HH203_RS15595	APC family serine transporter (YbeC-like)	
15	HH203_RS15615	Molybdenum cofactor biosynthesis protein F	**Cofactor and lipid biosynthesis module**
	HH203_RS15620	Aspartate aminotransferase HemL	
	HH203_RS15625	SDR oxidoreductase (3-oxoacyl-ACP reductase)	
16	sfnG	Dimethylsulfone monooxygenase SfnG	**Organosulphur compound and fatty acid oxidation module**
	HH203_RS15800	Acyl-CoA dehydrogenase family protein	
17	HH203_RS16095	HD domain phosphohydrolase (RpfG-like)	**c-di-GMP signalling two-component module**
	HH203_RS16100	GGDEF/EAL domain response regulator	
18	HH203_RS17170	Carboxynorspermidine decarboxylase	**Amino acid metabolism module**
	HH203_RS17175	Saccharopine dehydrogenase Lys9	
19	HH203_RS22195	ECF family RNA polymerase sigma factor	**ECF sigma-factor-mediated stress response module**
	HH203_RS22200	SRPBCC family ligand-binding protein YndB	
20	HH203_RS23520	LuxR C-terminal–related transcriptional regulator	**c-di-GMP signalling two-component module**
	HH203_RS23530	Bifunctional GGDEF/EAL/HAMP protein	
21	HH203_RS25285	Proline iminopeptidase-family hydrolase	**Peptidases and quorum-sensing-linked regulator module**
	HH203_RS25290	LuxR family transcriptional regulator SdiA-like	
	HH203_RS25295	Aminopeptidase P family protein PepP	
22	HH203_RS28595	HlyD family secretion protein EmrA-like	**ABC transporter-linked multidrug efflux**
	rbbA	RbbA ABC transporter/ATPase-permease	
23	tagQ	Type VI secretion (T6SS) lipoprotein TagQ (SlyB-like)	**T6SS and accessory regulation module**
	HH203_RS29115	SUMF1/EgtB/PvdO family nonheme iron enzyme	
	HH203_RS29120	ABC lipoprotein release permease LolC/LolE	
	HH203_RS29125	ABC lipoprotein export ATPase LolD	
	HH203_RS29130	Ser/Thr protein kinase PpkA (T6SS regulator)	
	HH203_RS29135	PP2C family protein phosphatase Stp1 (T6SS regulator)	
	tagF	TagF/ImpM family protein (T6SS regulator)	
	tssM	TssM inner membrane subunit/T6SS	
	HH203_RS29150	DotU/TssL-like protein/T6SS	
	tssK	TssK baseplate subunit/T6SS	
	tssJ	TssJ lipoprotein/T6SS	
	tagH	TagH FHA-domain protein/T6SS	
	tssA	TssA ATPase/assembly factor/T6SS	
	tssB	TssB contractile sheath small subunit/T6SS	
	tssC	TssC contractile sheath large subunit/T6SS	
	HH203_RS29185	Hcp family effector/T6SS	
	tssE	TssE baseplate subunit/T6SS	
	tssF	TssF baseplate subunit/T6SS	
	tssG	TssG baseplate subunit/T6SS	
	tssH	TssH ClpA-like ATPase/T6SS	
	HH203_RS29210	VgrG family protein/ T6SS spike effector protein	
	HH203_RS29215	DcrB-related protein	
	HH203_RS29220	RHS repeat/PAAR domain protein (T6SS tip)	
24	HH203_RS29505	ABC amino acid transporter SBP HisJ	**Polar amino acid ABC uptake module**
	HH203_RS29510	ABC amino acid permease HisM	
25	HH203_RS29715	CDP-alcohol phosphatidyltransferase PgsA	**Membrane phospholipid remodelling/biogenesis module**
	HH203_RS29720	Alpha/beta hydrolase and SAM-dependent methyltransferase PldB	
	HH203_RS29725	PAP2 family phosphatase	
	HH203_RS29735	Lysophospholipid acyltransferase PlsC	
	HH203_RS29740	Phosphatidate cytidylyltransferase (CDP-diglyceride synthase)	

* Gene symbols are provided instead of gene ID when available.

Several clusters encode metabolic or biosynthetic modules, including sulphur- and amino acid metabolism (Clusters 3 and 18), NAD biosynthesis/salvage (Cluster 7), acetoin/pyruvate dehydrogenase (Cluster 4), a cofactor and lipid biosynthesis (Cluster 15), organosulphur compound and fatty-acid oxidation (Cluster 16) and myo-inositol utilization and sugar ABC transport (Cluster 9). Within Cluster 9, an *iol* locus encodes for myo-inositol catabolism (iolC/E/B/I/D, an inositol dehydrogenase and ABC-type sugar transporters). Previous work in *Pseudomonas* has linked the *iol* locus to inositol-responsive behaviours, including fluorescent siderophore accumulation and competitive competence [43]. In our assays, all *Pseudomonas* strains grew in myo-inositol medium, even strains lacking the cluster (i.e. antimicrobial-negative strains), indicating that this locus is not necessarily required for myo-inositol catabolism but may be plausibly associated with inositol-responsive activity that influences the production or accumulation of antimicrobial molecules.

Transport and efflux systems were also prominent and included a cell-wall remodelling, metabolism and polyamine transport (Cluster 2), phosphate acquisition and multidrug efflux (Cluster 12), ABC transporter-linked multidrug efflux/secretion (Clusters 5 and 22), several amino acid and sugar ABC transporters (Clusters 9, 14 and 24), branched-chain amino acid transport with redox metabolism (Cluster 10) and a dedicated Type I secretion/efflux system (Cluster 13). Cluster 19 represents an ECF sigma-factor-mediated stress response. Cluster 21 links two peptidases with a LuxR-family regulator, suggesting peptide processing coupled to quorum-sensing-like regulatory control. Cluster 25 encodes key enzymes involved in membrane phospholipid biogenesis and remodelling, supporting membrane integrity. We also identified a subset of genomic regions (Clusters 1, 8 and 11) that remain largely uncharacterized, consisting mainly of DUF-containing or conserved hypothetical proteins without clear functional annotation, and therefore represent promising regions for future functional dissection of novel antimicrobial determinants.

Beyond these clusters, we also identified TCSs and c-di-GMP signalling systems (Clusters 6, 17 and 20) uniquely present in *B. glumae* antimicrobial-positive strains, supporting a role for signal transduction in regulating secretome-mediated antimicrobial activity. TCSs act as environmental signal transducers that enable bacteria to adapt to changing conditions and are recognized as broad regulators of antibiotic and secondary metabolite biosynthesis, thereby influencing biocontrol traits in beneficial bacteria such as *Pseudomonas*, *Bacillus* and *Streptomyces* [44–46]. Consistent with this, our comparative genomic analysis identified Clusters 6, 17 and 20 as enriched for TCS-related signalling and regulatory genes and further revealed that the response regulator HH203_RS27265 (*creC*), though located outside these clusters, represents an additional TCS as exclusively present in antimicrobial-positive strains. Interestingly, a deletion of *creC* in the antagonistic bacterium *Pseudomonas taiwanensis* reduces its antimicrobial activity against the rice pathogen *Xanthomonas oryzae* pv. *oryzae* (*Xoo*) [47].

Our most significant result was uncovering T6SS and an accessory regulation module within Cluster 23 exclusively present in antimicrobial-producing strains. Remarkably, we identified 23 T6SS-related genes, including those encoding structural proteins (TssA–TssM), baseplate components, Hcp, VgrG, a PAAR tip effector, lipoproteins and associated kinases/phosphatases [48]. Its completeness and regulatory composition support a potential role in interbacterial antagonism and delivery of antimicrobial molecules. Although T6SS is classically viewed as a contact-dependent interbacterial weapon [49], emerging research suggests broader roles in *Pseudomonas*, including links to the secretion and regulation of bioactive metabolites such as pyoverdine [50]. One possible hypothesis is that T6SS may indirectly influence secretome-mediated antimicrobial activity by shaping metabolic output, export or regulatory states that favour the accumulation of toxic or competitive metabolites in the extracellular milieu. Such coordination would allow antimicrobial-positive strains to deploy a contact-independent chemical offence while retaining the capacity for T6SS-mediated competition in natural communities. However, these relationships remain speculative and provide us with hypotheses to be tested with functional validation.

## Conclusion

The comparative genomics analyses conducted in this work uncovered 25 genomic clusters potentially associated with the antimicrobial activity of *P. protegens* PBL3. These clusters comprise metabolic, regulatory, secretion and transport systems besides biosynthetic pathways, suggesting that antimicrobial activities are multifactorial. These results highlight comparative genomics as a robust framework to identify genomic regions associated with antimicrobial activity to prioritize genes and pathways for future functional characterization. Overall, the results of this study provide an important foundation for future experimental validation of the 25 prioritized clusters, enabling the identification of antimicrobial-associated genomic regions and the determination of their specific mechanistic roles in the antimicrobial activity of *P. protegens* PBL3.

## Supplementary material

10.1099/mgen.0.001827 Supplementary Material 1.

10.1099/mgen.0.001827 Supplementary Material 2.

10.1099/mgen.0.001827 Supplementary Material 3.

10.1099/mgen.0.001827 Supplementary Material 4.

10.1099/mgen.0.001827 Supplementary Material 5.

10.1099/mgen.0.001827 Supplementary Material 6.

## Abbreviations

ANI: average nucleotide identity
antiSMASH: Antibiotics and Secondary Metabolite Analysis Shell
BCAs: biological control agents
BGCs: biosynthetic gene clusters
2,4-DAPG: 2,4-diacetylphloroglucinol
IGV: Integrative Genomics Viewer
KB: King’s B
KEGG: Kyoto Encyclopedia of Genes and Genomes
KO: KEGG Orthology
LCBs: locally collinear blocks
NRPS: non-ribosomal peptide synthetase
OD_600_: OD at 600 nm
TCSs: two-component systems
T6SS: Type VI secretion system
