# Supplementary material for: Comparative genomics approaches to identify genomic regions associated with the antimicrobial activity of Pseudomonas protegens PBL3
Source: Microb Genom. 2026 Sep 3;12(9):001827. doi: 10.1099/mgen.0.001827 (PMC13541209; doi:10.1099/mgen.0.001827)
Supplement: Supplementary file 1 [file mgen-12-01827-s001.pdf]

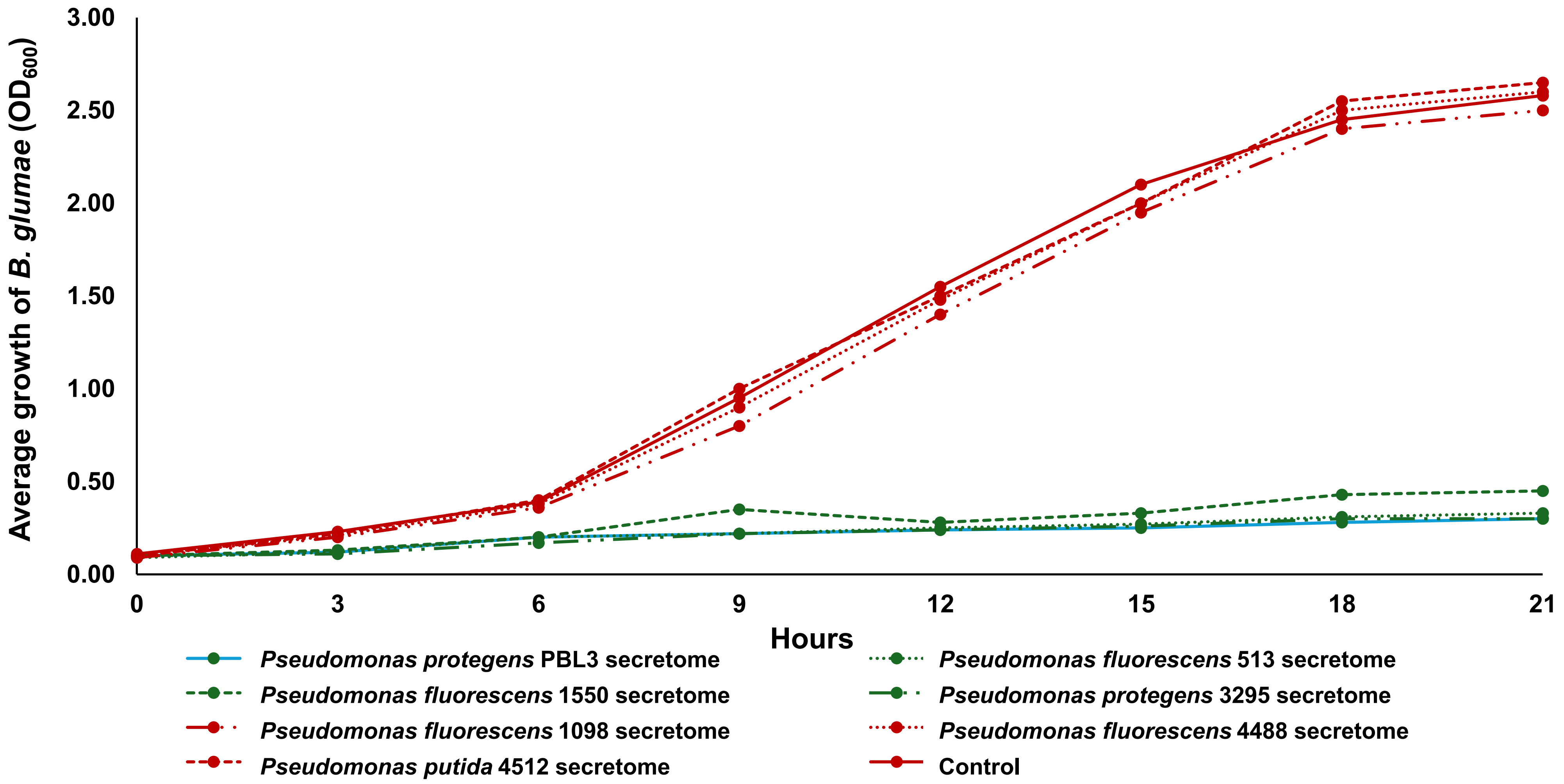

Supplementary Figure 1. Dynamic growth inhibition assay of *B. glumae* treated with secretomes from different *Pseudomonas* spp. strains.
B. glumae growth curve was evaluated in the presence of cell-free secretomes from different *Pseudomonas* spp. strains: *Pseudomonas protegens* PBL3 (blue solid line)*;* *Pseudomonas fluorescens* 513 (green dotted line); *Pseudomonas fluorescens* 1550 (green dashed line)*, Pseudomonas protegens* 3295 (green dash-dot line)*;* *Pseudomonas fluorescens* 1098 (red dash-dot line); *Pseudomonas fluorescens* 4488 (red dotted line)*; Pseudomonas putida* 4512 (red dashed line*);* and compared with growth in the absence of bacterial secretome (control, red solid line).

Supplementary Table 2A: Genome assembly statistics of *Pseudomonas* strains before scaffolding.

Assembly	*P. fluorescens* 1098	*P. fluorescens* 1550	*P. protegens* 3295	*P. fluorescens* 4488	*P. putida* 4512	*P. fluorescens* 513	*P. protegens* PBL3
Number of contigs	26	21	14	35	41	20	1
Total length	6302690	6565975	6907494	6068986	6730268	6354979	6847609
Largest contig	887834	1519658	1272117	798215	738760	1303717	6847609
GC content (%)	60.65	60.78	63.41	60.32	60.19	60.25	63.75
N50 (bp)	486653	644330	898942	380230	320058	665586	6847609
L50	5	3	4	6	8	4	1
Number of Ns per 100 kbp	0.00	0.00	2.82	0.00	0.00	7.74	4151.80

Supplementary Table 2B: Genome assembly statistics of *Pseudomonas* strains after scaffolding, using the *P. protegens* PBL3 genome as reference.

Assembly	*P. fluorescens* 1098	*P. fluorescens* 1550	*P. protegens* 3295	*P. fluorescens* 4488	*P. putida* 4512	*P. fluorescens* 513	*P. protegens* PBL3
Number of contigs	4	5	5	7	11	5	1
Total length	6304890	6567575	6908394	6071786	6733268	6356479	6847609
Largest contig	6297084	6547660	6891074	6012107	6113096	6346794	6847609
GC content (%)	60.65	60.78	63.41	60.32	60.19	60.25	63.75
N50 (bp)	6297084	6547660	6891074	6012107	6113096	6346794	6847609
L50	1	1	1	1	1	1	1
Number of Ns per 100 kbp	34.89	24.36	15.85	46.11	44.55	31.34	4151.8

Supplementary Table 2C: Genome assembly statistics of *Pseudomonas* strains after scaffolding, using the *P. protegens* CHA0 genome as reference.

Assembly	*P. fluorescens* 1098	*P. fluorescens* 1550	*P. protegens* 3295	*P. fluorescens* 4488	*P. putida* 4512	*P. fluorescens* 513	*P. protegens* CHA0
Number of contigs	4	5	5	6	11	6	1
Total length	6304890	6567575	6908394	6071886	6733268	6356379	6867980
Largest contig	6297084	6547660	6891074	6061642	6113096	6270881	6867980
GC content (%)	60.65	60.78	63.41	60.32	60.19	60.25	63.39
N50 (bp)	6297084	6547660	6891074	6061642	6113096	6270881	6867980
L50	1	1	1	1	1	1	1
Number of Ns per 100 kbp	34.89	24.36	15.85	47.76	44.55	29.77	0

Supplementary Table 6. KEGG based functional classification of candidate genes associated with antimicrobial activity.

Gene Identifier	KEGG Orthology (KO) identifiers with description	Metabolism	Enzyme class
HH203_RS12720	K06606 iolI; 2-keto-myo-inositol isomerase [EC:5.3.99.11]	Carbohydrate	Isomerases
HH203_RS12725	K03336 iolD; 3D-(3,5/4)-trihydroxycyclohexane-1,2-dione acylhydrolase (decyclizing) [EC:3.7.1.22]	Carbohydrate	Hydrolases
HH203_RS12730	K00010 iolG; myo-inositol 2-dehydrogenase / D-chiro-inositol 1-dehydrogenase [EC:1.1.1.18 1.1.1.369]	Carbohydrate, Biosynthesis of secondary metabolites	Oxidoreductases
HH203_RS12910	K16044 iolW; scyllo-inositol 2-dehydrogenase (NADP+) [EC:1.1.1.371]	Carbohydrate	Oxidoreductases
HH203_RS19700	K18369 adh2; alcohol dehydrogenase [EC:1.1.1.-]	Carbohydrate	
HH203_RS16150	K00209 fabV, ter; enoyl-[acyl-carrier protein] reductase / trans-2-enoyl-CoA reductase (NAD+) [EC:1.3.1.9 1.3.1.44]	Carbohydrate, Lipid	Oxidoreductases
HH203_RS00960	K00135 gabD; succinate-semialdehyde dehydrogenase / glutarate-semialdehyde dehydrogenase [EC:1.2.1.16 1.2.1.79 1.2.1.20]	Carbohydrate, Cofactors and vitamins, Amino acid	Oxidoreductases
HH203_RS14855	K00626 ACAT, atoB; acetyl-CoA C-acetyltransferase [EC:2.3.1.9]	Carbohydrate, Amino acid, Lipid, Energy, Terpenoids and polyketides, Xenobiotics biodegradation and metabolism	
HH203_RS12705	K03338 iolC; 5-dehydro-2-deoxygluconokinase [EC:2.7.1.92]	Carbohydrate	Transferases
HH203_RS12710	K03335 iolE; inosose dehydratase [EC:4.2.1.44]	Carbohydrate	Lyases
HH203_RS12715	K03337 iolB; 5-deoxy-glucuronate isomerase [EC:5.3.1.30]	Carbohydrate	Isomerases
HH203_RS12130	K08281 pncA; nicotinamidase/pyrazinamidase [EC:3.5.1.19 3.5.1.-]	Cofactors and vitamins	Hydrolases
HH203_RS00575	K00325 pntB; proton-translocating NAD(P)+ transhydrogenase subunit beta [EC:7.1.1.1]	Cofactors and vitamins	Translocases
HH203_RS07355	K00278 nadB; L-aspartate oxidase [EC:1.4.3.16]	Cofactors and vitamins, Amino acid	Oxidoreductases
HH203_RS12135	K00763 pncB, NAPRT1; nicotinate phosphoribosyltransferase [EC:6.3.4.21]	Cofactors and vitamins	Ligases
HH203_RS12140	K01916 nadE; NAD+ synthase [EC:6.3.1.5]	Cofactors and vitamins	Ligases
HH203_RS16650	K09458 fabF, OXSM, CEM1; 3-oxoacyl-[acyl-carrier-protein] synthase II [EC:2.3.1.179]	Cofactors and vitamins, Lipid	Transferases
HH203_RS27705	K00297 metF, MTHFR; methylenetetrahydrofolate reductase (NADH) [EC:1.5.1.54]	Cofactors and vitamins, Energy	Oxidoreductases
HH203_RS15620	K01845 hemL; glutamate-1-semialdehyde 2,1-aminomutase [EC:5.4.3.8]	Cofactors and vitamins	Isomerases

Gene Identifier	KEGG Orthology (KO) identifiers with description	Metabolism	Enzyme class
HH203_RS15625	K00059 fabG, OAR1; 3-oxoacyl-[acyl-carrier protein] reductase [EC:1.1.1.100]	Cofactors and vitamins, Lipid, Biosynthesis of secondary metabolites	Oxidoreductases
HH203_RS17170	K13747 nspC; carboxynorspermidine decarboxylase [EC:4.1.1.96]	Amino acid	Lyases
HH203_RS17175	K00290 LYS1; saccharopine dehydrogenase (NAD+, L-lysine forming) [EC:1.5.1.7]	Amino acid	Oxidoreductases
HH203_RS05585	K00003 hom; homoserine dehydrogenase [EC:1.1.1.3]	Amino acid	Oxidoreductases
HH203_RS25295	K08688 E3.5.3.3; creatinase [EC:3.5.3.3]	Amino acid	Hydrolases
HH203_RS01955	K04093 pheA1; chorismate mutase [EC:5.4.99.5]	Amino acid	Isomerases
HH203_RS12470	K01778 dapF; diaminopimelate epimerase [EC:5.1.1.7]	Amino acid	Isomerases
HH203_RS29735	K00655 plsC; 1-acyl-sn-glycerol-3-phosphate acyltransferase [EC:2.3.1.51]	Lipid	Transferases
HH203_RS29740	K00981 E2.7.7.41, CDS1, CDS2, cdsA; phosphatidate cytidylyltransferase [EC:2.7.7.41]	Lipid	Transferases
HH203_RS11265	K06998 phzF; trans-2,3-dihydro-3-hydroxyanthranilate isomerase [EC:5.3.3.17]	Biosynthesis of secondary metabolites	Isomerases
HH203_RS15795	K17228 sfnG; dimethylsulfone monooxygenase [EC:1.14.14.35]	Energy	Oxidoreductases
HH203_RS09540	K13481 xdhA; xanthine dehydrogenase small subunit [EC:1.17.1.4]	Nucleotide	Oxidoreductases
HH203_RS21210	K16842 hpxB; allantoinase [EC:3.5.2.5]	Nucleotide	Hydrolases
HH203_RS26235	K13789 GGPS; geranylgeranyl diphosphate synthase, type II [EC:2.5.1.1 2.5.1.10 2.5.1.29]	Terpenoids and polyketides	
HH203_RS10695	K01055 pcaD; 3-oxoadipate enol-lactonase [EC:3.1.1.24]	Xenobiotics biodegradation and metabolism	Hydrolases
HH203_RS10555	K15658 ofaA, arfA; arthrofactin-type cyclic lipopeptide synthetase A	Nonribosomal peptide synthetase	
HH203_RS07000	K24700 mdo; 3-mercaptopropionate dioxygenase [EC:1.13.11.91]		Oxidoreductases
HH203_RS02835	K18979 queG; epoxyqueuosine reductase [EC:1.17.99.6]		Oxidoreductases
HH203_RS12870	K20938 ladA; long-chain alkane monooxygenase [EC:1.14.14.28]		Oxidoreductases
HH203_RS25285	K18457 laaA; L-proline amide hydrolase [EC:3.5.1.101]		Hydrolases
HH203_RS29135	K20074 prpC, phpP; PPM family protein phosphatase [EC:3.1.3.16]		Hydrolases
HH203_RS15525	K01406 prtC; serralysin [EC:3.4.24.40]		Hydrolases
HH203_RS15570	K01266 dmpA, dap; D-aminopeptidase [EC:3.4.11.19]		Hydrolases
HH203_RS06935	K22278 pgdA; peptidoglycan-N-acetylglucosamine deacetylase [EC:3.5.1.104]		Hydrolases
HH203_RS09815	K03168 topA; DNA topoisomerase I [EC:5.6.2.1]		Isomerases
HH203_RS29130	K12132 prkC, stkP; eukaryotic-like serine/threonine-protein kinase [EC:2.7.11.1]		Transferases

Gene Identifier	KEGG Orthology (KO) identifiers with description	Genetic Information Processing
HH203_RS19600	K25495 nimR; AraC family transcriptional regulator, regulator of nimT	Transcription regulation
HH203_RS24780	K07506 K07506; AraC family transcriptional regulator	
HH203_RS03130	K25495 nimR; AraC family transcriptional regulator, regulator of nimT	
HH203_RS10675	K21405 acoR; sigma-54 dependent transcriptional regulator, acetoin dehydrogenase operon transcriptional activator AcoR	
HH203_RS15585	K14063 feaR; AraC family transcriptional regulator, positive regulator of tynA and feaB	
HH203_RS22195	K03088 rpoE; RNA polymerase sigma-70 factor, ECF subfamily	
HH203_RS25290	K07782 sdiA; LuxR family transcriptional regulator, quorum-sensing system regulator SdiA	
HH203_RS10895	K21919 KCTD9; BTB/POZ domain-containing protein KCTD9	Folding, sorting and degradation
HH203_RS17930	K03701 uvrA; excinuclease ABC subunit A	Replication and repair
**Gene Identifier**	**KEGG Orthology (KO) identifiers with description**	**Environmental Information Processing**
HH203_RS13535	K02040 pstS; phosphate transport system substrate-binding protein	ABC transporters
HH203_RS12740	K17213 K17213; inositol transport system substrate-binding protein	
HH203_RS12885	K01997 livH; branched-chain amino acid transport system permease protein	
HH203_RS01200	K02030 ABC.PA.S; polar amino acid transport system substrate-binding protein	
HH203_RS10855	K01992 ABC-2.P; ABC-2 type transport system permease protein	
HH203_RS12535	K15553 ssuA; sulfonate transport system substrate-binding protein	
HH203_RS12745	K10441 rbsA; ribose transport system ATP-binding protein [EC:7.5.2.7]	
HH203_RS12750	K10440 rbsC; ribose transport system permease protein	
HH203_RS12880	K01995 livG; branched-chain amino acid transport system ATP-binding protein	
HH203_RS12890	K01998 livM; branched-chain amino acid transport system permease protein	
HH203_RS12900	K01996 livF; branched-chain amino acid transport system ATP-binding protein	
HH203_RS13530	K02040 pstS; phosphate transport system substrate-binding protein	
HH203_RS28335	K11073 potF, spuD, spuE; putrescine transport system substrate-binding protein	
HH203_RS28595	K01993 ABC-2.TX; HlyD family secretion protein and efflux transporter periplasmic adaptor subunit	
HH203_RS28600	K01990 ABC-2.A; ABC-2 type transport system ATP-binding protein, rbbA	
HH203_RS29505	K02030 ABC.PA.S; polar amino acid transport system substrate-binding protein	
HH203_RS29510	K02029 ABC.PA.P; polar amino acid transport system permease protein	
HH203_RS14575	K11904 vgrG; type VI secretion system secreted protein VgrG	Type VI secretion system
HH203_RS29110	K06077 slyB; type VI secretion system-associated lipoprotein; tagQ	
HH203_RS29160	K11906 vasD, lip; type VI secretion system protein VasD; tssJ	

Gene Identifier	KEGG Orthology (KO) identifiers with description	Environmental Information Processing
HH203_RS29190	K11897 impF; type VI secretion system protein ImpF; tsssE	Type VI secretion system
HH203_RS29140	K11890 impM; type VI secretion system protein ImpM; tagF	
HH203_RS29145	K11891 impL, vasK, icmF; type VI secretion system protein ImpL; tssM	
HH203_RS29150	K11892 impK, ompA, vasF, dotU; type VI secretion system protein ImpK	
HH203_RS29155	K11893 impJ, vasE; type VI secretion system protein ImpJ; tssK	
HH203_RS29165	K11894 impI, vasC; type VI secretion system protein ImpI; tagH	
HH203_RS29170	K11902 impA; type VI secretion system protein ImpA; tssA	
HH203_RS29175	K11901 impB; type VI secretion system protein ImpB; tssB	
HH203_RS29180	K11900 impC; type VI secretion system protein ImpC; tssC	
HH203_RS29185	K11903 hcp; type VI secretion system secreted protein Hcp	
HH203_RS29195	K11896 impG, vasA; type VI secretion system protein ImpG; tssF	
HH203_RS29200	K11895 impH, vasB; type VI secretion system protein ImpH; tssG	
HH203_RS29205	K11907 vasG, clpV; type VI secretion system protein VasG; tssH	
HH203_RS29210	K11904 vgrG; type VI secretion system secreted protein VgrG	
HH203_RS10875	K07675 uhpB; two-component system, NarL family, sensor histidine kinase UhpB [EC:2.7.13.3]	Two-component system
HH203_RS10950	K20975 K20975; two-component system, sensor histidine kinase [EC:2.7.13.3]	
HH203_RS16070	K03406 mcp; methyl-accepting chemotaxis protein	
HH203_RS16095	K19623 phcQ; two-component system, probable response regulator PhcQ	
HH203_RS19645	K13587 cckA; two-component system, cell cycle sensor histidine kinase and response regulator CckA [EC:2.7.13.3]	
HH203_RS20575	K20973 sagS; two-component system, sensor histidine kinase SagS [EC:2.7.13.3]	
HH203_RS23520	K07684 narL; two-component system, NarL family, nitrate/nitrite response regulator NarL	
HH203_RS27265	K07641 creC; two-component system, OmpR family, sensor histidine kinase CreC [EC:2.7.13.3]	
HH203_RS11295	K02014 TC.FEV.OM; iron complex outer membrane receptor protein	Other transporters
HH203_RS11310	K05834 rhtB; homoserine/homoserine lactone efflux protein	
HH203_RS13075	K11688 dctP, yiaO; TRAP-type transport system periplasmic protein	
HH203_RS11205	K05517 tsx; nucleoside-specific channel-forming protein	
HH203_RS13810	K03561 exbB; biopolymer transport protein ExbB	
HH203_RS15835	K18138 acrB, mexB, adeJ, smeE, mtrD, cmeB; multidrug efflux pump	
HH203_RS22555	K03758 arcD, lysI, lysP; arginine:ornithine antiporter / lysine permease	
HH203_RS13550	K08152 lmrP; MFS transporter, DHA1 family, multidrug resistance protein B	Drug transporters
**Gene Identifier**	**KEGG Orthology (KO) identifiers with description**	**Environmental Information Processing**
HH203_RS19370	K19577 ydhP; MFS transporter, DHA1 family, inner membrane transport protein	Drug transporters
HH203_RS15550	K12537 hasE, prtE, rsaE, prsE, eexE; membrane fusion protein, type I secretion system	Type I secretion system

HH203_RS15555	K12536 hasD, prtD, aprD, rsaD, prsD, eexD; ATP-binding cassette, subfamily C, type I secretion system permease/ATPase	Type I secretion system
HH203_RS25640	K21498 higA-1; antitoxin HigA-1	Type II Toxin-Antitoxin system
**Gene Identifier**	**KEGG Orthology (KO) identifiers with description**	**Cellular Processes**
HH203_RS00815	K11931 pgaB; poly-beta-1,6-N-acetyl-D-glucosamine N-deacetylase [EC:3.5.1.-]	Biofilm formation
HH203_RS29545	K21084 yegE; diguanylate cyclase [EC:2.7.7.65]	
HH203_RS08465	K02556 motA; chemotaxis protein MotA	Flagellar system
HH203_RS28610	K02415 fliL; flagellar protein FliL	
**Gene Identifier**	**KEGG Orthology (KO) identifiers with description**	**Unclassified**
HH203_RS10685	K21416 acoA; acetoin:2,6-dichlorophenolindophenol oxidoreductase subunit alpha [EC:1.1.1.-]	
HH203_RS05910	K02426 sufE; cysteine desulfuration protein SufE	
HH203_RS10690	K21417 acoB; acetoin:2,6-dichlorophenolindophenol oxidoreductase subunit beta [EC:1.1.1.-]	
HH203_RS16130	K12945 nudK; GDP-mannose pyrophosphatase NudK [EC:3.6.1.-]	
